# Characterization of Callose Deposition and Analysis of the Callose Synthase Gene Family of *Brassica napus* in Response to *Leptosphaeria maculans*

**DOI:** 10.3390/ijms19123769

**Published:** 2018-11-27

**Authors:** Fei Liu, Zhongwei Zou, W. G. Dilantha Fernando

**Affiliations:** Department of Plant Science, University of Manitoba, Winnipeg, MB R3T 2N2, Canada; liuhuijuedui@163.com (F.L.); Zhongwei.Zou@umanitoba.ca (Z.Z.)

**Keywords:** *Brassica napus*, *Leptosphaeria maculans*, *CalS*, defense response, expression analysis

## Abstract

Callose plays a critical role in different biological processes including development as well as in the response to multiple biotic and abiotic stresses. In this study, we characterized the callose deposition in cotyledons of different *Brassica napus* varieties post-inoculated with different *Leptosphaeria maculans* isolates. Further, members of the callose synthase gene were identified from the whole genome of *B. napus* using the 12 *Arabidopsis thaniana* callose synthase protein sequences, and were then classified into three groups based on their phylogenetic relationships. Chromosomal location and duplication patterns indicated uneven distribution and segmental duplication patterns of *BnCalS* genes in the *B. napus* genome. Subsequently, gene structures, conserved domains analysis, and protein properties were analyzed for *BnCalS* genes. In addition, 12 *B. napus* orthologs of the *AtCalS* were selected for investigating the tissue expression pattern, indicating diverse expression patterns for these *BnCalS* genes. Responses of the selected 12 orthologs and all the *BnCalS* genes were characterized in the different types (*AvrLm1*-*Rlm1*, *AvrLm4*-*Rlm4*, *AvrLepR1*-*LepR1*) of *B. napus*–*L. maculans* interactions and *B. napus*-*Leptosphaeria biglobosa* interactions, implying their potential roles in response to *Leptosphaeria* infection.

## 1. Introduction

Blackleg, caused by *Leptosphaeria maculans* (*L. maculans*), is an economically important disease on *Brassica napus* (*B. napus*) and is common in North America, Australia, and Europe [1]. There are several measures to control this disease including crop rotation, fungicide application, seed treatments, and planting of resistant cultivars [2]. Using resistant cultivars is normally considered as an effective as well as an economic method of disease control. Regarding resistance for *L. maculans* in *Brassica* crops, qualitative loci related to seedling stage resistance and quantitative loci in resistance of adult plants have been reported by different groups [3]. So far, a total of 18 qualitative loci have been reported, including *Rlm1*, *Rlm2* [4], *Rlm3*, *Rlm4* [5], *Rlm5*, *Rlm6* [6,7], *Rlm7* [5], *Rlm8*, *Rlm9* [5,8], *Rlm10* [9], *Rlm11* [7,10], *LepR1* and *LepR2* [11,12], *LepR3* [13,14], *LepR4* [15], and *RlmS* [16].

Callose is a polysaccharide existing widely in the cell walls of a variety of higher plants. Its major composition is β-1,3-glucan. Synthesis and degradation of callose are involved in a variety of processes in plant development as well as in responses to multiple biotic and abiotic stresses [17]. Callose has been proposed to play crucial roles in another development. Its synthesis and degradation are tightly controlled during and following meiosis [18]. Callose is believed to be deposited as a temporary callose wall between the primary cell wall and the plasma membrane, preventing microsporocytes fusion and cohesion as well as maintaining the microsporocyte morphology and shielding microsporocytes from the influence of the surrounding environment [19,20]. Callose is synthesized by callose synthases (CalS) [21,22], most of which play vital roles in diverse biological processes [22,23]. *CalS7* plays a role in plant growth and reproduction [24]. Many reports revealed that synthesis of callose was influenced by abiotic and biotic stresses, including injury, low temperature, heavy metals, and various pathogens. *GSL5*/*Cals12* was found to be responsible for callose formation at sites of pathogen attack and at wounding sites [25,26,27]. In addition, expression of five callose synthase genes (*AtCalS1*, *5*, *9*, *10*, and 12) was induced by the pathogen (*Hyaloperonospora arabidopsis*) or salicylic acid (SA) [28]. In *Citrus limon*, increased susceptibility to *Xanthomonas citrisubsp. citri* was caused by the silencing of *ClCalS1* [29]. It was shown that down-regulation of *HvGsl6*, an orthologue of *AtGsl5* (*Cals12*) in barley, leads to lower accumulation of papillary and wound callose as well as a higher susceptibility to the pathogen *Blumeria graminis* f. sp. *hordei* [30].

*B. napus* (AACC, 2*n* = 38) is an allotetraploid species that was formed by hybridization between ancestors of *Brassica oleracea* (*B. oleracea*, CC, 2*n* = 18) and *Brassica rapa* (*B. rapa*, AA, 2*n* = 20) [31], followed by allopolyploidy [32]. Rapeseed/canola oil occupies the third position of oil production, after palm and soybean oils [33]. However, the production of rapeseed/canola is facing various threats, especially from diseases. A better understanding of the genes involved in defense responses will be helpful in breeding for the control of diseases. While little has been explained about callose deposition and the callose synthase gene family in *B. napus*, especially in response to blackleg, the *CalS* gene family plays important roles in different plant physiological processes. In this study, we characterized the callose deposition in two different *R*-*Avr* interactions, and subsequently identified 32 *CalS* genes in *B. napus* by protein-protein Basic Local Alignment Search Tool (BLASTP) against the *B. napus* genome using protein sequences of 12 *CalS* genes from *Arabidopsis thaliana* (*A. thaliana*). The phylogenetic relationships of *CalS* genes between *B. napus*, *B. rapa*, *B. oleracea*, and *A. thaliana* were analyzed, and gene structures, conserved domains, protein properties, and duplication patterns were all analyzed. Subsequently, selected *BnCalS* gene family members or all the members were revealed for tissue expression patterns and their response to *L. maculans* or *L. biglobosa* isolates in different varieties.

## 2. Results

### 2.1. Characterization of Callose Deposition in B. napus with the Infection by L. maculans Isolates

**Figure 1:**
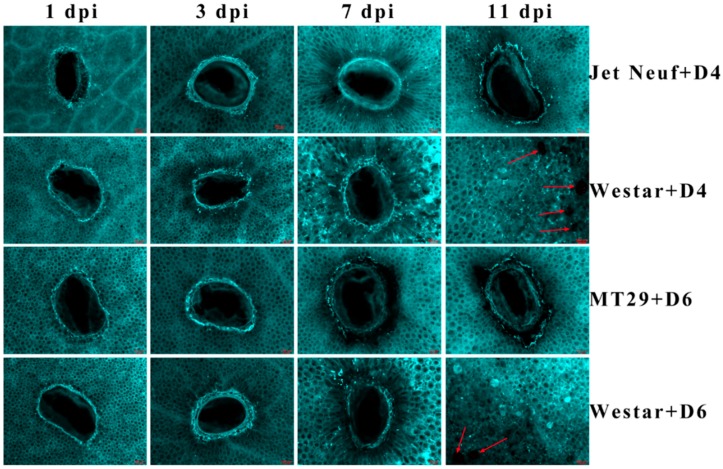
Staining of callose deposition in different *B. napus* varieties inoculated with different *L. maculans* isolates. Jet Neuf + D4 and MT29 + D6 indicate cotyledons of Jet Neuf, and MT29 were inoculated with D4 and D6 isolates, respectively. Westar + D4 and Westar + D6 indicate cotyledons of Westar were inoculated with D4 and D6 isolates, respectively. One, 3, 7 and 11 dpi indicate 1, 3, 7 and 11 days post-inoculation. Red arrows indicate the pycnidia produced by *L. maculans*. Three replicates were performed for each treatment and showed a similar pattern. Scale bars = 100 μm.

Callose was suggested to have a vital role in response to plant pathogens. In order to test the alteration of callose deposition in different types (*AvrLm1*-*Rlm1*, *AvrLm4*-*Rlm4*) of *B. napus*–*L. maculans* interactions, aniline blue dye was used to stain cotyledons of different *B. napus* varieties (with *Rlm1*, *Rlm4*, or no *R* gene) inoculated with different *L. maculans* isolates. As shown in Figure 1, all the cotyledons displayed a circle of staining near the inoculation sites 1-day post inoculation. Unconsolidated distribution of callose appeared in the cotyledons of the susceptible cultivar Westar, which were inoculated with D4 or D6 isolates and observed for 3, 7 or 11 days. In contrast, the incompatible interactions, Jet Neuf with D4 isolate and MT29 with D6 isolate, showed a pyknotic style of callose distribution around the infection sites through the whole infection process (Figure 1). However, deposition was continuous along with the whole infection process during the compatible interactions (Westar with D4 or D6, Figure 1).

### 2.2. Genome-Wide Identification of BnCals Family Genes from B. napus

**Figure 2:**
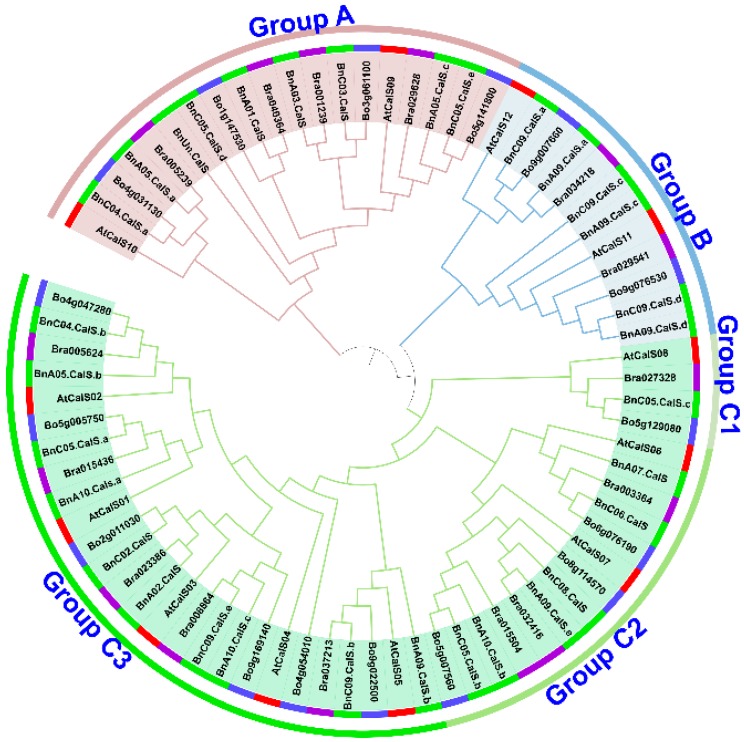
Phylogenetic relationship analysis of *CalS* genes in *A. thaliana*, *B. napus*, *B. rapa*, and *B. oleracea*. Protein sequences of twelve AtCalS from *A. thaliana*, 32 BnCalS from *B. napus*, 15 BrCalS from *B. rapa*, and 16 BoCalS from *B. oleracea* were used to construct a neighbor-joining (NJ) tree. Different colors of the first circle from the tree represent different genes from different species, with red indicating *A. thaliana*, green indicating *B. napus*, purple representing *B. rapa*, and skyblue denoting *B. oleracea*. The second circle of the tree indicates three groups or five subgroups differentiated by different colors.

Using 12 *A. thaliana* CalS protein sequences as queries, thirty-two *BnCalS* genes were identified and validated via reciprocal BLASTP between the genome database for *B. napus* and *A. thaliana*. The length of BnCalS proteins varied from 603 to 2085 aa, and the corresponding molecular weights of the BnCalS proteins ranged from 69.11 to 241.01 kDa (Table 1). The isoelectric point (pI) values of the BnCalS proteins were all higher than seven (Table 1), indicating the partial alkaline properties of BnCalS proteins. Transmembrane domain analysis showed that BnCalS proteins all contain several transmembrane domains, with values from 8 to 20 (Table 1). The protein sequences of *BnCalS* genes, *AtCalS* genes, and their homologs in *B. rapa* and *B. oleracea* were also used for constructing phylogenetic trees to analyze the evolutionary relationships of CalS proteins. In total, 32 BnCalS, 12 AtCalS, 15 BrCalS, and 16 BoCals proteins were used for generating the phylogenetic tree (Figure 2). As shown in Figure 2, *CalS* gene families were divided into three groups: Group A, Group B and Group C, which were subsequently divided into three subgroups: Group C1, Group C2 and Group C3. Group A and Group B contain 9 and 6 *BnCalS* genes, respectively (Table 1), while 1, 6 and 10 BnCalS members were included in Group C1, C2, and C3, respectively (Table 1).

### 2.3. Chromosomal Localization and Duplication of BnCalS Gene Family in B. napus

**Figure 3:**
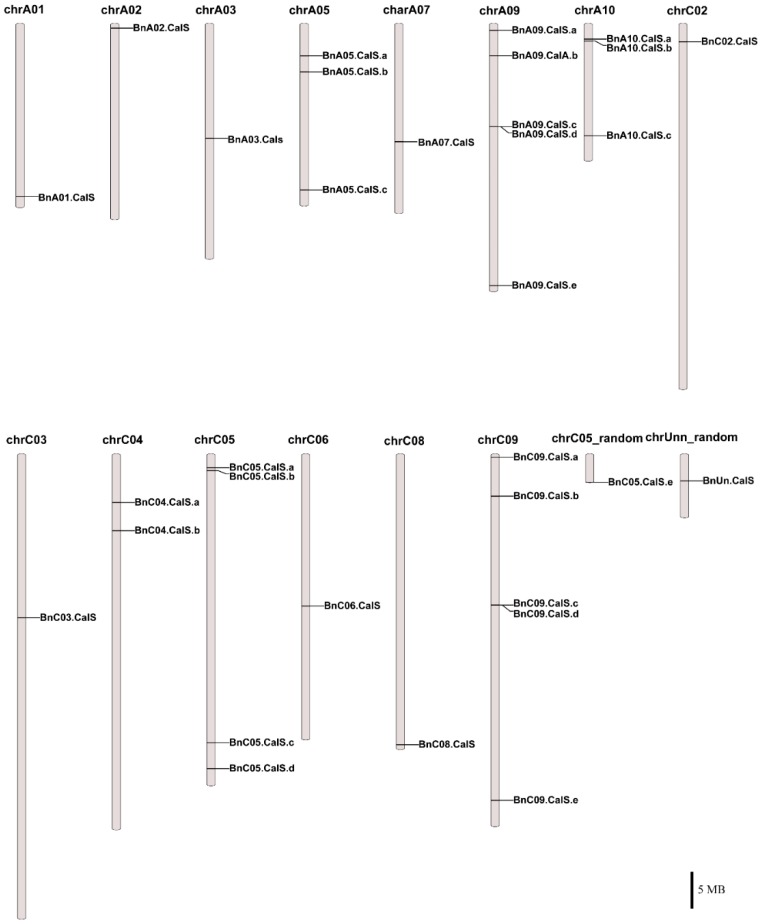
Chromosomal locations of 32 *BnCalS* genes. The chromosome number is placed above each chromosome. Only those chromosomes containing *BnCalS* genes (15) are listed. Scale bar represents a 5 Mb physical distance.

Thirty-two *BnCalS* genes were mapped onto 15 chromosomes in *B. napus* (Figure 3, Table S1). *BnCalS* genes were unevenly distributed throughout the chromosomes. Chromosome A09, C05, and C09 contained the maximum number of *CalS* genes (five), while some chromosomes contained no *CalS* genes, such as A04, A06, A08, C01 and C07 (Figure 3 and Table S1). Two chromosomes (A05 and A10) contained three *CalS* genes (Figure 3 and Table S1) and chromosome C04 contained two *CalS* genes (Figure 3 and Table S1). Nine chromosomes, A01, A02, A03, A07, C02, C03, C06, C08 and chrUnn_random, contained only one *CalS* gene (Figure 3 and Table S1).

**Figure 4:**
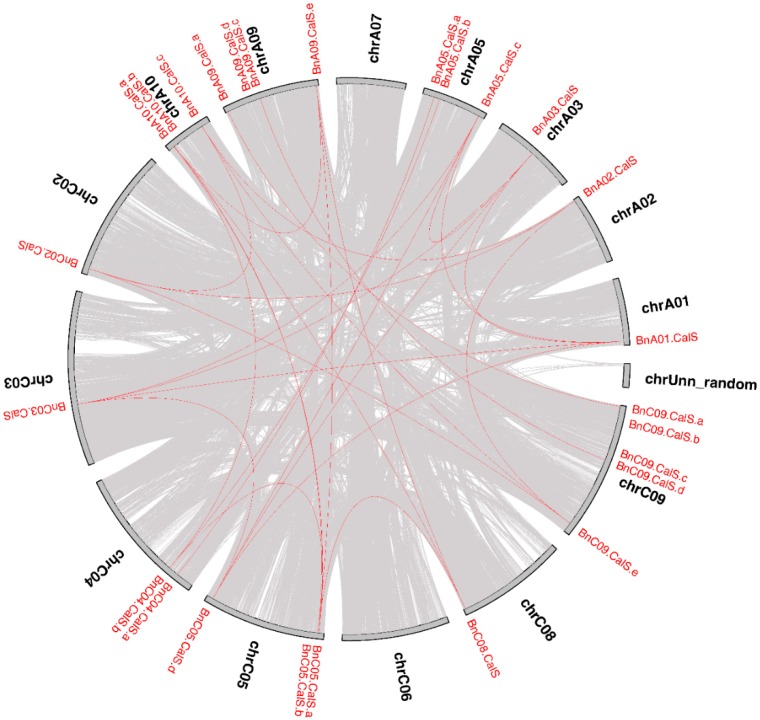
Circle plot showing collinear gene pairs on 12 *Brassica napus* chromosomes. Grey lines represent collinear blocks in the whole *B. napus* genome, while red lines indicate collinear *BnCalS* gene pairs.

Several gene duplication modes including whole-genome duplication (WGD) or segmental duplication, tandem duplication, and rearrangements were considered to drive the evolution of protein-coding gene families at the gene and chromosomal level [34]. The contribution of the *CalS* gene family to the expansion of the *B. napus* genome was investigated using MCscanX software suite [35]. According to a whole genome analysis of gene duplications, two types of duplication modes, dispersed and WGD/segmental, were detected among 32 *BnCalS* genes (Table S1). Twenty-seven *BnCalS* genes were produced by WGD, with five others produced by dispersed duplications (Table S1). The collinear relationships of the duplicated pairs in the *BnCalS* gene family in *B. napus* were investigated using MCscanX and are shown in Figure 4. In total, we identified 30 paralog pairs that shared a higher identity according to their protein sequences (Figure 4). Enormous synteny blocks were also identified between A and C subgenomes or between different chromosomes of A or C, and all the 30 paralog pairs were located within synteny blocks on chromosomes (Figure 4).

### 2.4. Gene Structure and Conserved Domain Analysis of BnCalS Genes

**Figure 5:**
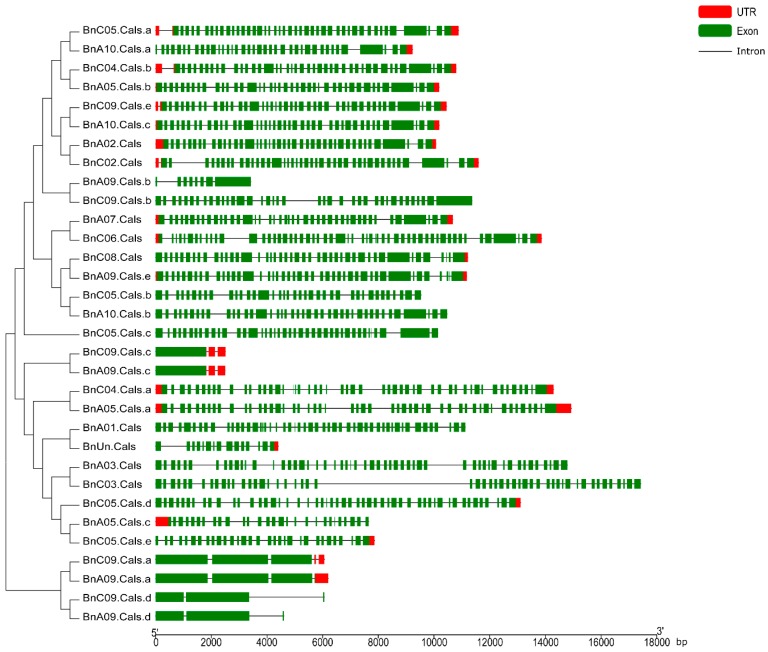
Gene structures of the *BnCalS* genes according to their phylogenetic relationships. The red boxes and green boxes indicate untranslated regions (UTR) and exons, respectively, black lines denote introns. bp: base pairs.

**Figure 6:**
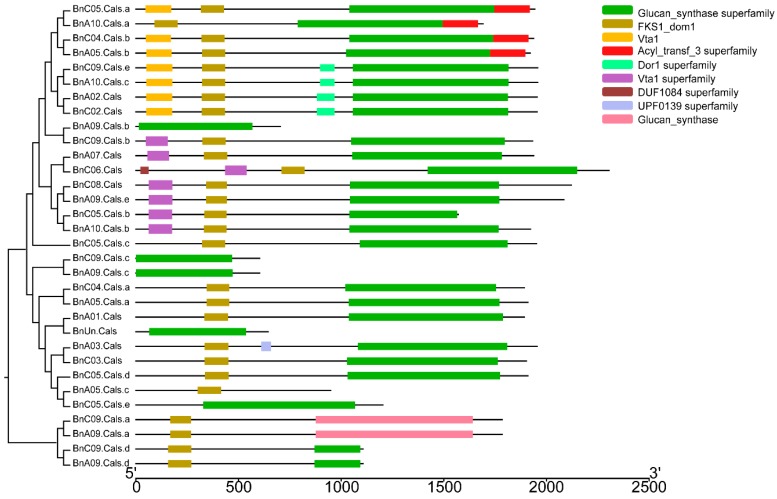
Schematic representation of the conserved domains in the *BnCalS* genes. Each predicted domain is represented by a colored box indicated at the right side.

The open reading frame (ORF) length of the 32 *BnCalS* genes ranged from 1809 to 6912 bp, with an average of 4977 bp (Table 1). The number of exons in the *BnCalS* gene family varied from one to 55, with most genes (29 of 32, 71.9%) having more than 30 exons (Figure 5 and Table S1). The gene pairs among the *BnCalS* gene family shared a high similarity in terms of exon numbers and protein/ORF length (Figure 5 and Table S1). For example, the gene pairs *BnA01.Cals*: *BnC03.Cals* had 50 and 50 exons, respectively, and gene pair *BnA09.Cals.d*: *BnC09.Cals.d* had 3 and 3 exons, respectively (Figure 5 and Table S1). The conserved domains in *BnCalS* gene family were investigated using Batch CD-Search. Nine conserved domains were discovered in the 32 *CalS* genes, and the Glucan_synthase superfamily domain was observed to be the most common domain (30 of 32) in the BnCalS proteins, followed by FKS1_dom1 domain, which was found to be in 27 BnCalS proteins (Figure 6). However, the domains DUF1084 superfamily and UPF0139 superfamily were observed in only one protein, BnC06.CalS and BnA03.CalS, respectively (Figure 6).

### 2.5. Expression Analysis of the BnCalS Gene Family in Different Tissues

**Figure 7:**
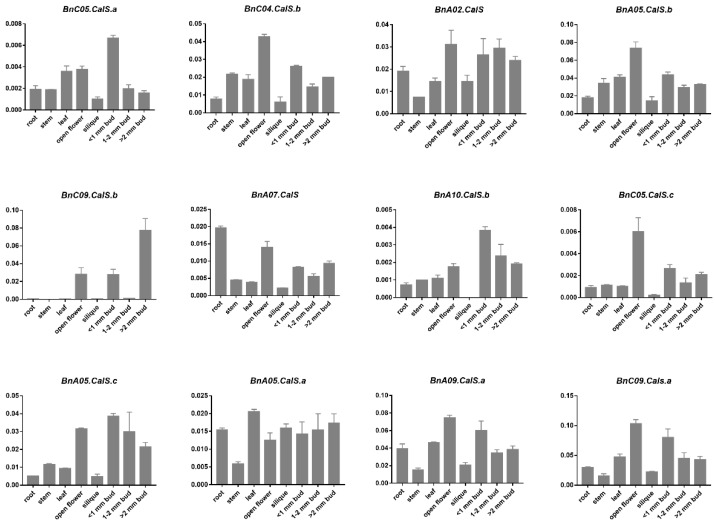
Expression analysis of 12 selected *BnCalS* genes in different tissues and at different developmental stages. The different tissues included root, stem, leaf, silique, and bud as indicated in the figure. <1 mm bud, 1–2 mm bud, and >2 mm bud indicate buds with different sizes, with diameters >1 mm, 1–2 mm, or >2 mm. Expression was detected using qPCR. The values and error bars indicate means ± standard error (*n* = 3).

To gain a better understanding of the functions of *BnCalS* genes in plant development, we selected 12 *BnCalS* genes showing the highest similarity with 12 *AtCalS* genes and analyzed their expression patterns in six tissues including root, stem, leaf, flower, silique, and bud at three different developmental stages (<1 mm, 1–2 mm, >2 mm) using quantitative RT-PCR (qPCR) (Table S2). We found that the *BnCalS* genes had various kinds of expression patterns (Figure 7); *BnC09.Cals.b* showed expression only in open flowers and buds, *BnA10.CalS.b* had no expression in silique. The remaining nine *BnCalS* genes displayed expression in all the six tissues (Figure 7). Most of the *BnCalS* genes exhibited a higher expression level in open flowers and buds (Figure 7), suggesting key roles of *BnCalS* genes in the flower development process. *BnA02.CalS* and *BnA05.CalS.a* displayed a higher expression level in all six tissues. Some *BnCalS* genes exhibited tissue-specific expression; *BnA07.CalS* was highly expressed in roots and open flowers, while *BnC05.CalS.c*, *BnC04.CalS.b*, and *BnA05.CalS.b* were strongly expressed in open flowers (Figure 7). In the three bud stages, *BnA02.CalS* had a higher expression level across all three bud stages, and *BnC05.CalS.a* was mainly expressed in the <1 mm stage, while *BnC09.CalS.b* showed higher expression levels in the >2 mm stage (Figure 7). Due to the low expression level detected here, we performed another cycle of qPCR using another known internal gene *GAPDH* (*BnaC05g09880D*) to validate the expression of *BnCalS* genes in different tissues. Most of these genes showed very similar expression patterns, as in Figure 7, except that expression levels increased a little in most genes (Figures S3 and S4).

### 2.6. Expression Analysis of BnCalS Genes in Different R-Gene-Containing Varieties Infected by L. maculans Isolates with Different Avr Genes

**Figure 8:**
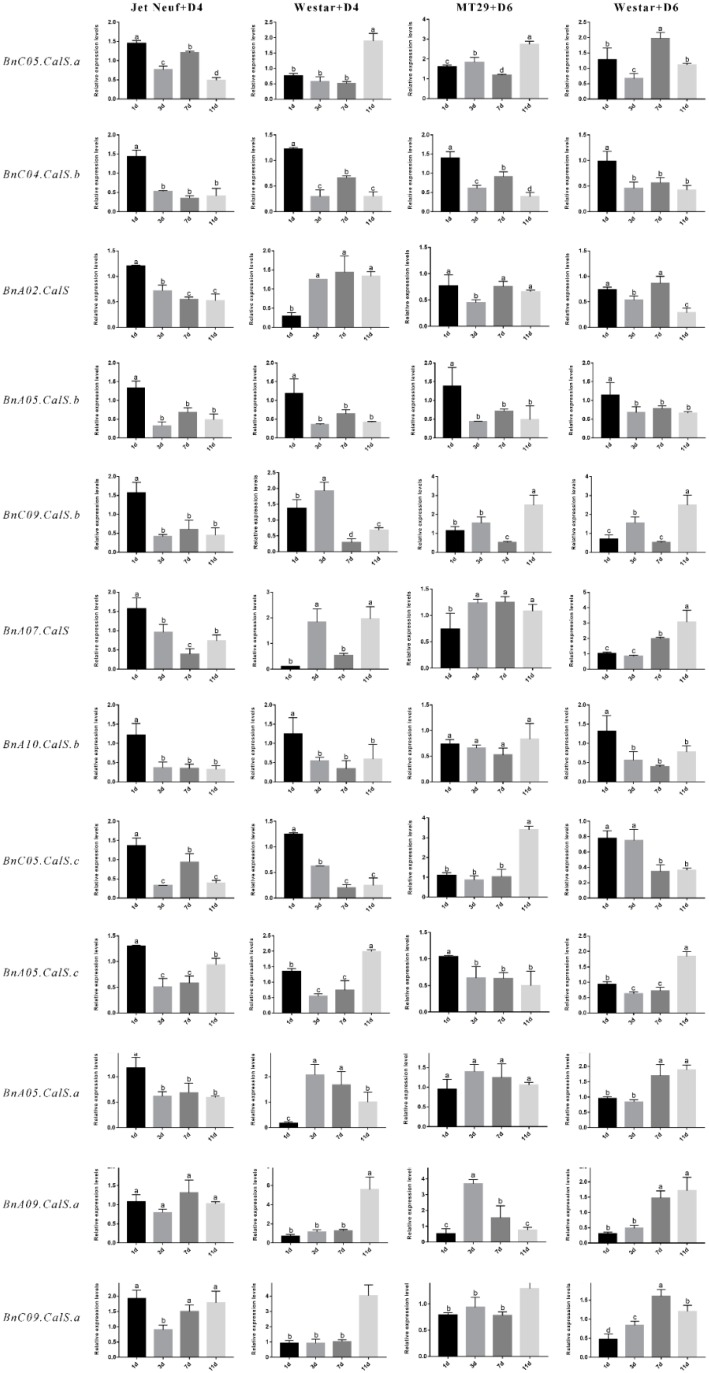
Expression analysis of selected 12 *BnCalS* genes in two *B. napus* varieties following inoculation with *Leptosphaeria maculans*. Three different varieties, Westar, MT29, and Jet Neuf, were inoculated with two *L. maculans* isolates, D4 and D6, and expression of the corresponding 12 *CalS* genes at different time points was determined using qPCR. The *Brassica napus Actin* gene was used as the internal control to normalize expression data. The results were calculated from three biological replicates. Tukey’s test (ANOVA) were performed to determine the significant difference between each treatment. The statistically significant differences were defined as *p* < 0.05 and are marked with different letters. Error bars denote standard error.

Callose synthesis was reported to be involved in defense against different kinds of pathogens [36,37,38,39,40]. To gain insight into the roles of *BnCalS* genes in the responses of *B. napus* against *L. maculans*, 12 *BnCalS* with higher similarity to 12 *AtCalS* genes were selected for determining expression levels in varieties of MT29 (containing *Rlm1* and *Rlm9*, inoculated with D6), Jet Neuf (containing *Rlm4*, inoculated with D4), and Westar (containing no R gene, inoculated with D4 or D6). One-week-old cotyledons from each cultivar were punctured prior to inoculation. As shown in Figures S1 and S2, the interactions of MT29 with D6, and Jet Neuf with D4, were defined as incompatible interactions, while interactions of Westar with D6 or D4 were indicated as compatible interactions. The expression of the 12 selected *BnCalS* genes showed diverse patterns (Figure 8). There were two genes, *BnC04.CalS.b* and *BnA05.CalS.b*, the expressions of which were shown to be suppressed under four treatments (Figure 8). Expression of the twelve selected genes appeared to decrease or not be altered in Jet Neuf after inoculation with D4 isolate (Figure 8). However, when the cultivar Westar was inoculated with D4 isolate, eight of the 12 *BnCalS* genes, *BnC05.CalS.a*, *BnA02.CalS*, *BnC09.Cals.b*, *BnA07.CalS*, *BnA05.Cals.c*, *BnA05.CalS.a*, *BnA09.CalS.a*, and *BnC09.CalS.a*, increased their expression (Figure 8). In the cultivar MT29, expression of *BnC05.CalS.a*, *BnC09.CalS.b*, *BnA07.CalS*, *BnC05.CalS.c*, *BnA09.CalS.a*, and *BnC09.CalS.a* were induced by the infection of D6 isolates. When Westar was inoculated with D6 isolate, expressions of *BnC05.CalS.a*, *BnC09.CalS.b*, *BnA07.CalS*, *BnA05.CalS.c*, *BnA05.CalS.a*, *BnA09.CalS.a*, and *BnC09.CalS.a* were shown to be up-regulated after inoculation at different periods (Figure 8).

**Figure 9:**
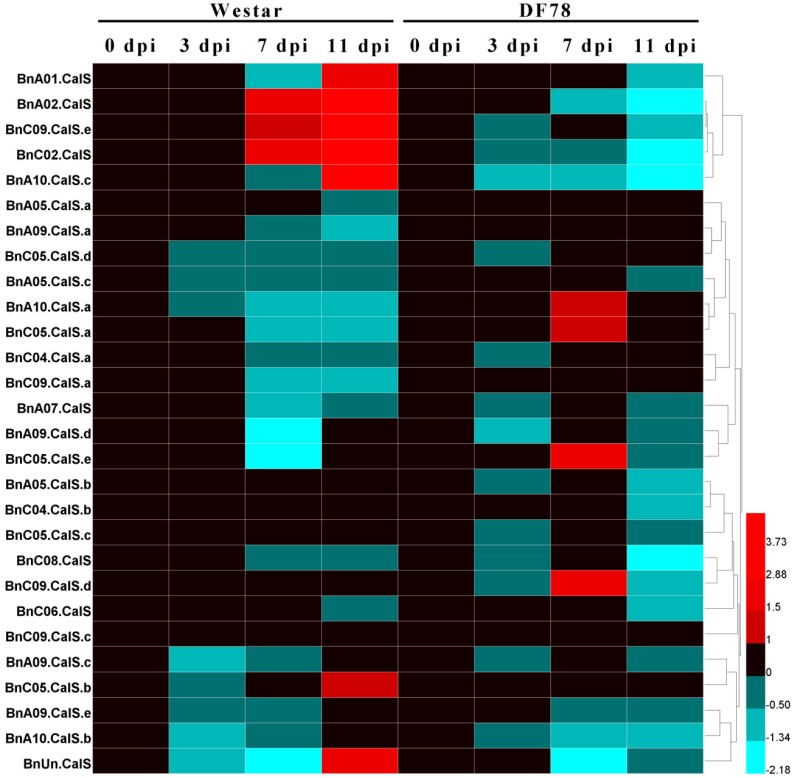
Heatmap of the expression patterns of the 28 *BnCalS* genes in Westar and DF78 after inoculation with D3 isolate for different time periods. Expression levels were measured in transcripts per kilobase of exon model per million mapped reads (TPM) values, ratio values were from comparing the TPM value of the *L. maculans* treated sample with that of the mock-treated sample. The color scale bar at the bottom of the map represents a log2 ratio value, which represents the low and high expression, respectively. Varieties and corresponding treatments used for expression profiling are indicated at the top of each column. The names of genes are indicated on the left side of the heatmap.

To further explore *BnCalS* gene expression in other types of *B. napus*–*L. maculans* pathosystems, such as the *AvrLepR1*–*LepR1* interaction or the *B. napus*–*L. biglobosa* pathosystem, we analyzed the expression level of these *BnCalS* genes under different types of pathosystems based on published RNA-seq datasets [41,42]. For analyzing the expression of *BnCalS* during the *AvrLepR1*–*LepR1* interaction, one-week-old seedlings of cultivars DF78 and Westar inoculated with of D3 isolates were harvested at different time points for RNA purification and subsequently RNA sequencing [42]. As shown in Figure 9, 11 of the 32 genes (4 of them were not shown because of low or no expression) were shown to be induced by the infection of *L. maculans* isolate D3 in both *B. napus* lines, Westar, and DF78. However, none of these genes were induced in both varieties: *BnA01.CalS*, *BnA02.CalS*, *BnC09.CalS.e*, *BnC02.CalS*, *BnA10.CalS.c*, *BnC05CalS.b*, and *BnUn.CalS* only increased their levels in Westar, while *BnA10.CalS.a*, *BnC05.CalS.a*, *BnC05.CalS.e*, and *BnC09.CalS.d* were only induced in DF78 (Figure 9). Expression levels of all the four up-regulated genes peaked at 7-days post-inoculation in DF78 (Figure 9). All the remaining *BnCalS* genes, except *BnA05.CalS.b*, *BnC04.CalS.b*, *BnA05.CalS.c*, *BnC09.CalS.c*, *BnA05*.CalS.*a*, and *BnA09.CalS.a*, showed repressed expression in both varieties (Figure 9). Expression of *BnA05.CalS.b*, *BnC04.CalS.b*, and *BnA05.CalS.c* were not altered in Westar but repressed in DF78, whereas, expression of *BnC09.CalS.a* and *BnA05.CalS.a* were displayed inversely (Figure 9). *BnC09.CalS.c* was not affected in both varieties (Figure 9).

### 2.7. Differential Expression Analysis of BnCalS Genes in the Interaction of B. napus with Two Leptosphaeria Species: L. maculans and L. biglobosa

**Figure 10:**
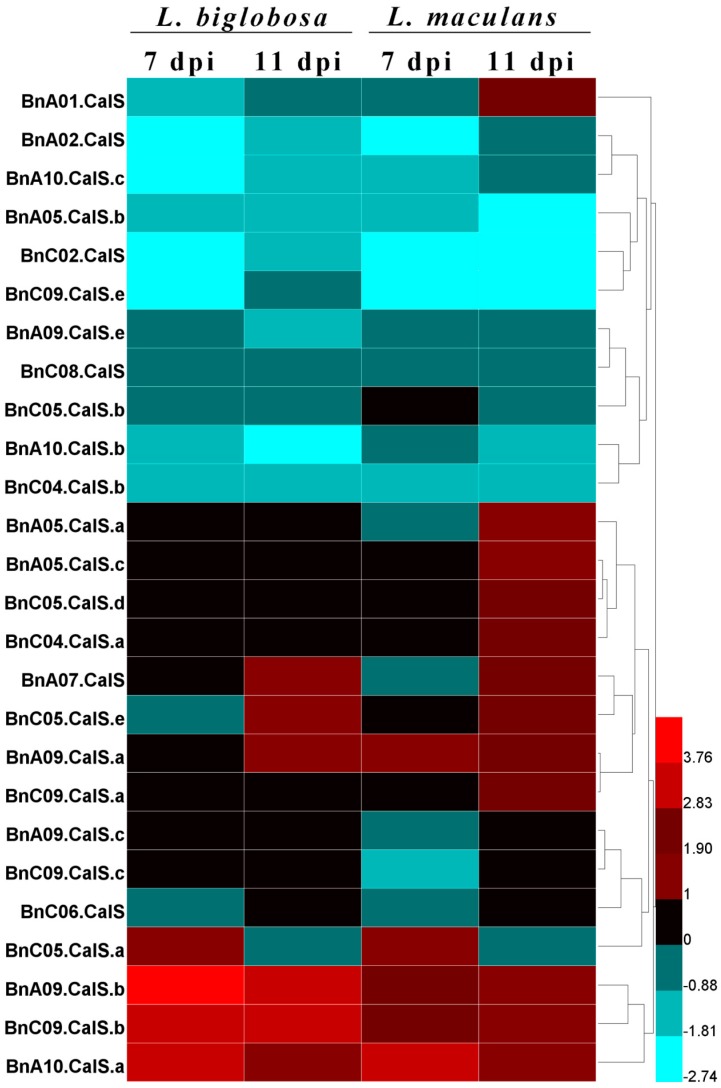
Heapmap of the expression profiles of *BnCalS* genes in Westar plants subjected to *L. biglobosa* or *L. maculans* infection. The TPM value of each gene was used as the expression level, the relative value was obtained by comparing TPM values of Westar inoculated with *L. maculans* or *L. biglobosa* with Westar inoculated with water, and the color scale was based on the log2 (relative value). Samples with *L. maculans* or *L. biglobosa* are indicated on the top of the heatmap, and genes are indicated at the left side of the heatmap. Twenty-six genes were shown and the other 6 were not included because of low or no expression in these samples.

*Leptosphaeria biglobosa* is closely related with *L. maculans*, both belonging to the *Leptosphaeria* genus [1,43,44]. As *L. biglobosa* is less aggressive and causes lower losses to oilseed rape production compared with *L. maculans*, it is essential to investigate the host response differences to these two species. We utilized the published RNA sequencing data [41] to differentiate the expression difference of *BnCalS* in Westar with inoculation by *L. maculans* and *L. biglobosa*. As stated by Lowe et al. [41], 10-day-old cotyledons of Westar were inoculated with *L. maculans* isolate IBCN18 and *L. biglobosa* isolate 06J154 and collected for RNA sequencing at 7 and 11 dpi, respectively. As shown in Figure 10, various kinds of expression patterns were indicated. The first class were those having ultra-low or no expression levels in the interaction of *B. napus* with both *Leptosphaeria* species, and those genes including *BnA03.CalS*, *BnA09.CalS.d*, *BnC03.CalS*, *BnC05.CalS.c*, *BnC09.CalS*.d, and *BnUn.CalS*. The second class were those whose expression was repressed in the interaction of Westar with both species, including *BnA02.CalS*, *BnA10.CalS.c*, *BnA05.CalS.b*, *BnC02.CalS*, *BnC09.CalS.e*, *BnA09.CalS.e*, *BnC08.CalS*, *BnC05.CalS.b*, *BnA10.CalS.b*, *BnC04.CalS.b*, and *BnC06.CalS*. The third class included *BnA05.CalS.a*, *BnA05.CalS.c*, *BnC05.CalS.d*, *BnC04.CalS.a*, and *BnC09.CalS.a*, in which the expression level was not altered in Westar inoculated with *L. biglobosa* but showed an elevated level in Westar inoculated with *L. maculans*. The fourth class was those which showed increased expression level in both kinds of interactions, and included *BnA07.CalS*, *BnC05.CalS.e*, *BnA09.CalS.a*, *BnC09.CalS.a*, *BnC05.CalS.a*, *BnA09.CalS.b*, *BnC09.CalS.b*, and *BnA10.CalS.a*. The fifth class included *BnA09.CalS.c* and *BnC09.CalS.c*, in which the expression was not altered in the Westar-*L. biglobosa* interaction but was repressed in the Westar-*L. maculans* interaction.

## 3. Discussion

Callose is suggested to have diverse functions during the process of plant development as well as in responses to environmental biotic or abiotic stimulus [17,22]. Blackleg is one of the major diseases around the world, and callose deposition plays an important role in the interaction of *B. napus* and *L. maculans* [42], the causal agent of blackleg. Here, we characterize the deposition of callose during the whole infection process of blackleg in *B. napus* using two different *R*-*Avr* interaction types (*Rlm1*-*AvrLm1*, *Rlm4*-*AvrLm4*). This contributed to knowledge of callose deposition during blackleg infection. During the incompatible interaction, pyknotic callose structure circled the infection site, which can be beneficial in enhancing the resistance of host to pathogen by preventing the extension of hyphae [45]. In the compatible interaction, unconsolidated distribution of callose around the infection site starting from 3 dpi may cause the *B. napus* to be more susceptible to *L. maculans*. However, callose production was continuous in compatible interactions, and higher after 7 dpi, which may suggest that callose production is a sign for responses of *B. napus* to *L. maculans*, and *R* genes could effectively modulate the callose deposition to form a compact callose wall around the infection site. Furthermore, behavior of callose deposition displays a very similar pattern between different types of *R*-*Avr* interactions, which might suggest that they share a similar regulation mechanism of callose deposition.

Callose is synthesized by callose synthases in plants, and 12 callose synthases genes were proposed in Arabidopsis [22]. Some of the 12 *AtCalS* genes were investigated for their functions in plant development [20,24,46,47] and defense against biotic or abiotic stress [28,45]. However, little is known about the *CalS* gene family in *B. napus*.

In this study, 32 *CalS* genes were identified in *B. napus*, which is almost three times the number of Arabidopsis *CalS* genes. This may suggest that genome duplication occurred in the evolution of *B. napus*, in accordance with the fact that *B. napus* is an allotetraploid species with widespread genome duplication and merging events [32]. *B. napus* contains the same number of genes as the total number of *B. rapa* and *B. oleracea*, which are two ancestral species of *B. napus* [31], indicating that *CalS* genes may be derived from *B. rapa* and *B. oleracea CalS* genes. Combing phylogeny with gene structures and protein properties, it was found that most of the genes in the same groups showed similar exon numbers and protein pI values, such as the group B members having 1–4 exons with pI values around 9, while most members of group A had approximately 50 exons with pI values about 8. This indicated that group classification based on the phylogenetic tree was supported by gene structure and protein character. Five gene duplication modes including whole-genome duplication (WGD) or segmental duplication, tandem duplication, and rearrangements were considered as the major driving forces for the evolution of gene families at the gene [34]. In addition, segmental gene duplication is one of the main reasons for maintenance of gene families [48]. Thirty-two *BnCalS* genes were unevenly distributed on 15 of 19 chromosomes, and 27 of them were found to be produced by segmental duplication, indicating that segmental duplication played an indispensable role in the evolution of the *CalS* gene family in *B. napus*. Based on a constructed phylogenetic tree, the 32 BnCalS proteins were classified into three groups, which showed similar patterns as reported previously [49]. In addition, the Glucan_synthase family or Glucan_synthase domain was detected in all the *BnCalS* gene family, indicating that these CalS proteins may have glucan synthase activity, which has the main role in callose synthesis.

Expression patterns of *CalS* genes in different tissues have not been reported for many plants. For this study, we selected the 12 *BnCalS* genes showing the most similarity with the 12 *AtCalS* genes for analyzing expression in seven different tissues. The qPCR results from two reference genes showed a small difference in the expression level, which might be mainly due to relatively stable expression but not absolutely constant expression of these reference genes. Homologs from different species usually share a similar function in the biological process. *BnC09.CalS.b* showed a relatively higher level in open flowers and buds, especially in buds with diameters larger than 2mm, which is recognized as the microspore stage [50]. This expression pattern is in accordance with that of *AtCalS5*, however, *BnC09.CalS.b* was not expressed in roots, leaves, stems, and silique, which is totally different from *AtClaS5*. For this case, we infer that different paralogs of *BnC09.CalS.b* may be expressed in these tissues to ensure the normal synthesis of callose, or that the other *CalS* family members may help to achieve normal formation of callose in these tissues. Callose is ultra-important for pollen development and entry into the ovary [17,20,24,51], and results from the present study also suggest that most of the *CalS* genes display a higher expression level in the open flower and bud development process, which indicate that these *CalS* genes play key roles in the process of pollen development and pollen grain germination. In addition, the 12 tested *CalS* showed different levels in different tissues, suggesting the complex expression profiles of the *CalS* gene family members in plant development stages, and that different members may be responsible for the synthesis of callose in different tissues and development stages.

Callose is considered to provide mechanical support to the cell wall against environmental stresses, and its synthesis can be altered by the infection of pathogens [45]. Callose was also found to be involved in the *B. napus*–*L. maculans* interaction [42]. Comparing the expression of *CalS* genes in different *B. napus* varieties inoculated with different isolates, it was found that most of the *BnCalS* genes were repressed in the varieties, suggesting that *L. maculans* may have mechanisms to regulate the expression of *CalS* genes. There are some *BnCalS* genes that displayed different expression patterns in response to different types of variety–isolate interactions; for instance, the expression of *BnC05.CalS.a* was suppressed in both DF78 and Westar inoculated with D3 isolate, while its expression was inhibited in Jet Neuf but activated in Westar when they were both inoculated with the D4 isolate. Such *BnCalS* genes like *BnC05.CalS.a* may be modulated by the interaction of specific *R* and *Avr* genes. Expression of some *BnCalS* genes, such as *BnC09.CalS.b*, were repressed in the susceptible variety of Westar but were induced in resistant varieties DF78 and MT29, which may contribute to resistance conferred by *R* genes. There are some genes whose expression peaked earlier in incompatible interactions of plant–pathogens, contributing to the prompt response to *L. maculans* infection, such as *BnA03.CalS* and *BnA09.CalS.d* in the interactions of DF78 and D3. Based on these results, we concluded that *BnCalS* plays a key role in the response of *B. napus* against *L. maculans* infection through mediating the synthesis of callose. In addition, more up-regulated *BnCalS* genes were found in the interaction of *B. napus*–*L. maculans*, indicating a stronger defense response. All up-regulated *BnCalS* genes found in the interaction of *B. napus*–*L. biglobosa* are also shared with up-regulated genes in the interaction of *B. napus*–*L. maculans*. This may indicate that these shared *BnCalS* genes are in the common signaling pathway for defense against *Leptosphaeria* species.

## 4. Materials and Methods

### 4.1. Plant and Fungal Materials

The susceptible *B. napus* cultivar Westar (no *R* gene) was inoculated with *L. maculans* isolate D4 or D6, while the *B. napus* lines Jet Neuf (*Rlm4*) and MT29 (*Rlm1* and *Rlm9*) were inoculated with *L. maculans* isolates D4 and D6, respectively. The D4 isolate contained seven avirulent genes *AvrLm4*, *AvrLm5*, *AvrLm6*, *AvrLm7*, *AvrLm8*, *AvrLepR1*, and *AvrLepR2*, and the D6 isolate contained five avirulent genes *AvrLm1*, *AvrLm5*, *AvrLm6*, *AvrLm8*, and *AvrLmS* [52]. *B. napus* seeds were germinated in soil and grown for one week, with a photoperiod of 16 h/8 h (16 °C dark, 22 °C light). The *L. maculans* inoculation was performed as described by Zhang et al. [52] and Liban, et al. [53]. Sterilized distilled water was used as mock control. Samples were collected and flash-frozen in liquid nitrogen. Cotyledons inoculated with *L. maculans* isolate or mock were sampled at the 1st day, 3rd day, 7th day, and 11th day after inoculation. Roots of Westar seedlings were used for root tissue, and stems of two-month-old Westar plants were used for stem tissue. For leaf tissue collection, the fifth leaves of 8-leaf-stage Westar plants were sampled. Buds with different diameters (<1 mm, 1–2 mm, and >2 mm) were collected from Westar plants and used for different stages of buds. Flowers were sampled the same day they bloomed. Silique samples were collected after flowering for two weeks.

### 4.2. Aniline Blue Staining of Callose Deposition

Callose deposition was stained using aniline blue according to the protocol by Schenk and Schikora [54]. Briefly, post-inoculated cotyledons were collected and fixed in formalin-acetic-acid-alcohol (FAA) solution (ethanol: acetic acid: formaldehyde: H_2_O = 10:1:2:7, *v*/*v*/*v*/*v*). Before staining, cotyledons were de-stained in 1:3 acetic acid/ethanol until they became transparent. Cotyledons were then washed three times using 150 mM K_2_HPO_4_, and washed cotyledons were immersed in staining solution and stained for at least 2 hrs. The staining solution was prepared by dissolving 0.01% aniline blue in 150 mM K_2_HPO_4_. Callose depositions were visualized on a Zeiss Axio Imager Z1 using a 4’,6-diamidino-2-phenylindole (DAPI) filter.

### 4.3. Identification of CalS in B. napus, B. rapa, and B. oleracea

The 12 AtCalS protein sequences from the *A. thaliana* genome (http://www.arabidopsis.org/) were used as queries to identify CalS genes in *B. napus*, *B. rapa*, and *B. oleracea* via a BLASTP search [55]. The BLASTP searches used default parameters, with E-value less than 1 × 10^−20^ and the score set at more than 1000. In addition, obtained BnCalS sequences were used as queries in BLASTP against the *A. thaliana* protein database for confirmation. The sequences of obtained proteins were analyzed using the Batch CD-Search (https://www.ncbi.nlm.nih.gov/Structure/bwrpsb/bwrpsb.cgi) to ensure target proteins contained glucan_synthase or glucan_synthase family domains. The identified CalS protein sequences of *B. napus*, *B. rapa*, *B. oleracea*, and *A. thaliana* were used for multiple sequence alignments using the ClustalW program. Phylogenetic relationships were established using alignment results with MEGA 6.0 [56] based on the neighbor-joining (NJ) method using a boot strap replication of 1000.

### 4.4. Chromosomal Location and Orthologous Identification of CalS

Chromosomal position information for BnCalS genes was retrieved from relevant generic feature format (GFF) files, which were downloaded from Ensembl plants. These *BnCalS* genes were denominated based on their position on the chromosomes. Gene duplication types and collinearity relationships were detected using MCscanX. BLASTP was performed to do self-self-comparison using *B. napus* protein sequences with the E-value under 1 × 10^−10^. Program detect_collinearity_within_gene_families.pl in MCscanX was used to detect collinearity within the *BnCalS* gene family [35]. Five duplication types including WGD/segmental duplication events were detected for classifying origins of the duplicate genes of the B. napus genome using the program incorporated in MCScanX, and duplication information of the *BnCalS* gene family was extracted. The duplications within the *BnCalS* gene family were displayed using the program circle_plotter of MCScanX.

### 4.5. Gene Structures and Protein Conserved Domains Analysis of the BnCalS Gene Family

The information of exon-intron structures for *BnCalS* genes was extracted from GFF files and displayed using TBtools software [57]. Conserved domains of the BnCalS proteins were investigated with the Batch CDD tool (https://www.ncbi.nlm.nih.gov/Structure/bwrpsb/bwrpsb.cgi) with default parameters used and displayed by TBtools.

### 4.6. RNA Purification and Quantitative RT-PCR Analysis

Total RNA from different tissues or cotyledons with different treatments was purified with PureLink™ Plant RNA Reagent (Invitrogen, Carlsbad, CA, USA). The purified RNA was treated with the TURBO DNA-free™ Kit (Invitrogen) for 30 minutes to degrade genomic DNA. Following the manufacturer’s instructions, the first-strand cDNA was synthesized using 1 μg of the total RNA with the 1st cDNA Synthesis Kit (Thermo Scientific, Waltham, MA, USA). For the qPCR assay, cDNA was diluted 1:100 with ddH_2_O, and 4.2 μL of cDNA plus 5 μL of SYBR Green I Master Mix (Clontech, Palo Alto, CA, USA) and 0.4 μL of each primer (10 mM) were used for the PCR reaction. PCRs were performed using a CFX96 Real Time Instrument (Bio-Rad, Hercules, CA, USA), and the amplification programs were set at the following conditions: 95 °C for 1 min, 40 cycles of 95 °C for 10 s, 60 °C for 30 s, followed by 95 °C for 10 min. Melting curve analysis was conducted by increasing 0.5 °C at 5 s/step from 65 to 95 °C to estimate the specificity of product. The relative gene expression levels of the target genes were calculated using the 2^−ΔΔ*C*t^ method with the *B. napus Actin* gene (AF111812.1) as a reference. All the primers used for qPCR were deposited in Table S2.

### 4.7. Transcriptome Analysis of BnCalS Genes Based on Published RNA-Seq Data

To investigate the expression patterns of *BnCalS* genes in the *AvrLepR1*-*LepR1* interaction system, Sequence Read Archive (SRA) files were downloaded from Gene Expression Omnibus (GEO) RNA-seq datasets (GSE77723). All reads were cleaned using trimmomatic 0.36 [58] and aligned to the transcriptome of *B. napus* ‘Darmor-bzh’ with Bowtie2 [59]. Gene expression quantification was performed using a software package called RSEM [60]. The transcripts per kilobase of exon model per million mapped reads (TPM) values were extracted from the expression quantification data, after which heatmaps were constructed from relative TPM values using HemI software (1.0) [61].

## Figures and Tables

**Table 1:** Protein information of *BnCalS* gene family in *Brassica napus*. ORF stands for open reading frame. aa indicates the length of the protein. pI denotes isoelectric point.

Gene Name	Locus Name	Group	ORF Length	aa	Molecular Weight	pI	No. Transmembrane Domains
*BnA01.Cals*	BnaA01g32440D	A	5676	1892	217.01 kDa	8.2	16
*BnA02.Cals*	BnaA02g01360D	C3	5862	1954	225.77 kDa	9.35	13
*BnA03.Cals*	BnaA03g29890D	A	5865	1955	224.00 kDa	8.75	14
*BnA05.Cals.a*	BnaA05g07560D	A	5730	1910	218.75 kDa	8.5	16
*BnA05.Cals.b*	BnaA05g11020D	C3	5763	1921	221.74 kDa	9.52	15
*BnA05.Cals.c*	BnaA05g30410D	A	5730	1910	218.93 kDa	8.61	16
*BnA07.Cals*	BnaA07g18170D	C2	5814	1938	225.22 kDa	9.36	12
*BnA09.Cals.a*	BnaA09g01630D	B	5352	1784	206.97 kDa	9.43	12
*BnA09.Cals.b*	BnaA09g08360D	C3	2112	704	80.55 kDa	9.79	9
*BnA09.Cals.c*	BnaA09g20050D	B	1809	603	69.11 kDa	9.92	9
*BnA09.Cals.d*	BnaA09g20060D	B	3318	1106	128.92 kDa	8.94	8
*BnA09.Cals.e*	BnaA09g49880D	C2	6255	2085	241.01 kDa	8.56	15
*BnA10.Cals.a*	BnaA10g03730D	C3	5073	1691	195.72 kDa	9.19	14
*BnA10.Cals.b*	BnaA10g04230D	C2	5766	1922	223.47 kDa	8.94	14
*BnA10.Cals.c*	BnaA10g20270D	C3	5871	1957	226.05 kDa	9.27	15
*BnC02.Cals*	BnaC02g04450D	C3	5865	1955	225.89 kDa	9.38	13
*BnC03.Cals*	BnaC03g35120D	A	5706	1902	217.82 kDa	8.93	14
*BnC04.Cals.a*	BnaC04g08410D	A	5679	1893	216.76 kDa	8.29	16
*BnC04.Cals.b*	BnaC04g12720D	C3	5811	1937	224.01 kDa	9.55	15
*BnC05.Cals.a*	BnaC05g03680D	C3	5829	1943	225.06 kDa	9.4	14
*BnC05.Cals.b*	BnaC05g04460D	C2	4710	1570	182.96 kDa	8.19	8
*BnC05.Cals.c*	BnaC05g39020D	C1	5856	1952	226.47 kDa	8.33	14
*BnC05.Cals.d*	BnaC05g44800D	A	3609	1203	137.48 kDa	8.1	11
*BnC05.Cals.e*	BnaC05g52230D	A	2847	949	110.38 kDa	7.19	5
*BnC06.Cals*	BnaC06g17090D	C2	6912	2304	266.81 kDa	8.91	20
*BnC08.Cals*	BnaC08g44800D	C2	6363	2121	245.25 kDa	8.91	15
*BnC09.Cals.a*	BnaC09g00800D	B	5352	1784	207.37 kDa	9.58	10
*BnC09.Cals.b*	BnaC09g08620D	C3	5799	1933	221.65 kDa	9.25	13
*BnC09.Cals.c*	BnaC09g22380D	B	1809	603	69.10 kDa	9.92	9
*BnC09.Cals.d*	BnaC09g22390D	B	3318	1106	129.01 kDa	9	8
*BnC09.Cals.e*	BnaC09g44080D	C3	5871	1957	226.02 kDa	9.27	15
*BnUn.Cals*	BnaUnng02530D	A	1935	645	72.56 kDa	9.12	8

## Supplementary Materials

Supplementary materials can be found at http://www.mdpi.com/1422-0067/19/12/3769/s1.
