# Supplementary material for: Characterization of Callose Deposition and Analysis of the Callose Synthase Gene Family of Brassica napus in Response to Leptosphaeria maculans
Source: Int J Mol Sci. 2018 Nov 27;19(12):3769. doi: 10.3390/ijms19123769 (PMC6320764; doi:10.3390/ijms19123769)
Supplement: Supplementary file 1 [file ijms-19-03769-s001.zip › title.pdf]

Table S1: Chromosomal information of the *BnCalS* gene family and their duplication modes in *Brassica napus*.

Table S2: Sequences of primers used for quantitative RT- PCR of *BnCalS* genes.

Figure S1: Disease symptoms of two *Brassica napus* varieties, MT29 (*Rlm1* and *Rlm9*) and Westar (no *R*), inoculated with *Leptosphaeria maculans* D6 (*AvrLm1-5-6-8-S*).

Figure S2: Disease symptoms of two *Brassica napus* varieties, Jet Neuf (*Rlm4*) and Westar (no *R*), inoculated with *Leptosphaeria maculans* D4 (*AvrLm4-5-6-7-8-LepR1-LepR2*).

Figure S3: Relative expression levels of the selected 12 *BnCalS* genes in eight different tissues or developmental stages using the *GAPDH* gene as reference gene.

Figure S4: Gel electrophoresis of qPCR amplicons of eight different tissues or developmental stages. A-E indicate amplification from roots, stems, leaves, open flowers, silique, <1 mm bud, 1-2 mm bud, and >2 mm bud, respectively. The *BnGAPDH* gene was used as the reference gene.
