# Supplementary figures and images for: Characterization of Callose Deposition and Analysis of the Callose Synthase Gene Family of Brassica napus in Response to Leptosphaeria maculans
Source: Int J Mol Sci. 2018 Nov 27;19(12):3769. doi: 10.3390/ijms19123769 (PMC6320764; doi:10.3390/ijms19123769)

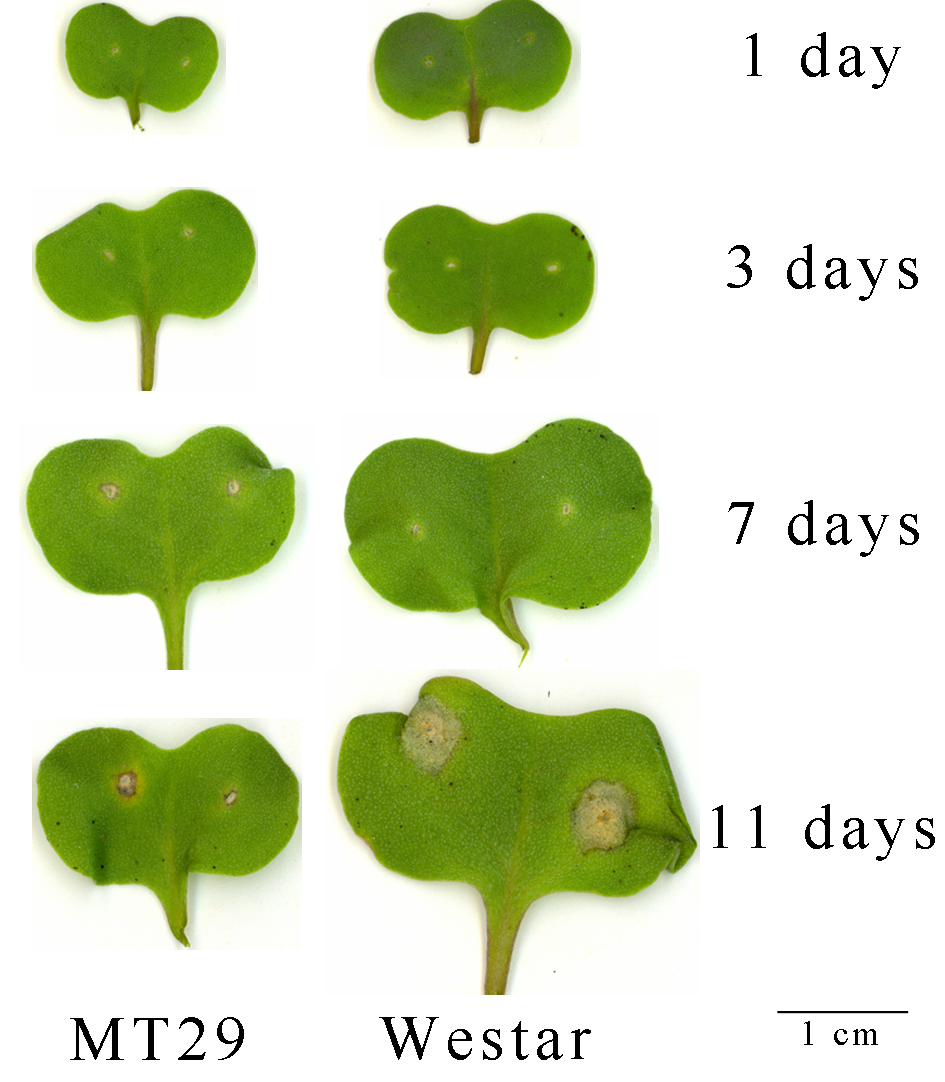

Supplement: Supplementary file 1 [file ijms-19-03769-s001.zip › Figure S1.tif]

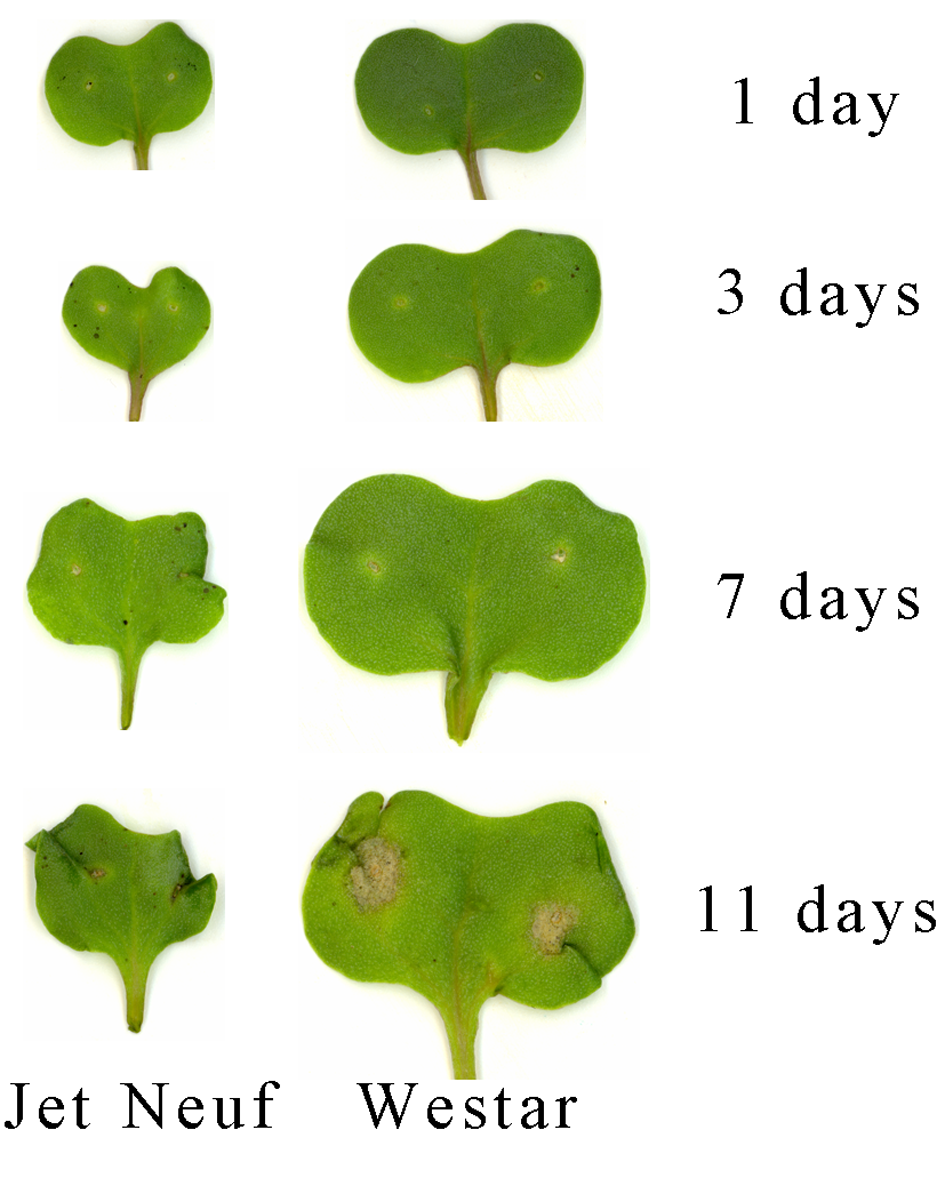

Supplement: Supplementary file 1 [file ijms-19-03769-s001.zip › Figure S2.tif]

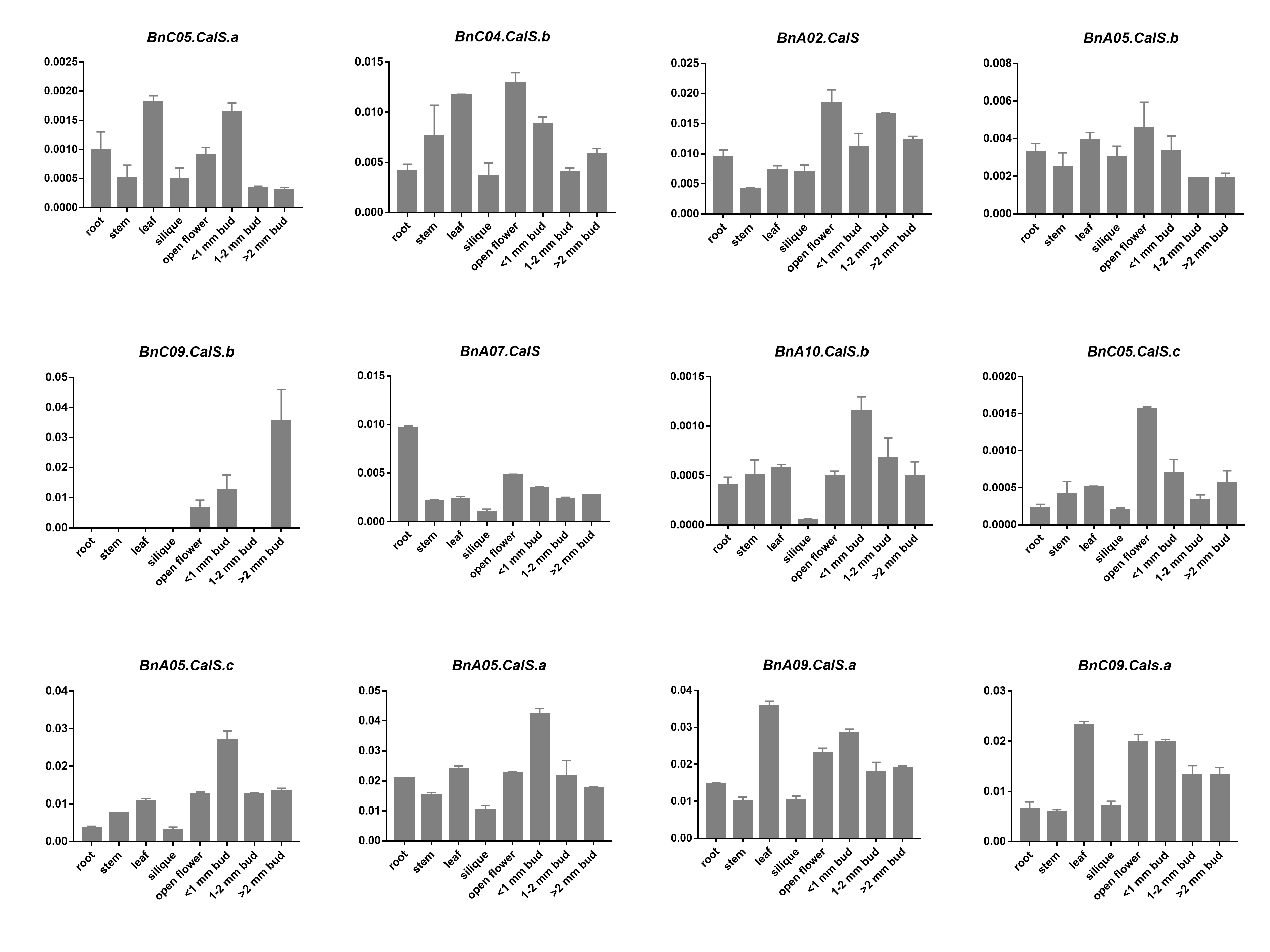

Supplement: Supplementary file 1 [file ijms-19-03769-s001.zip › Figure S3.tif]

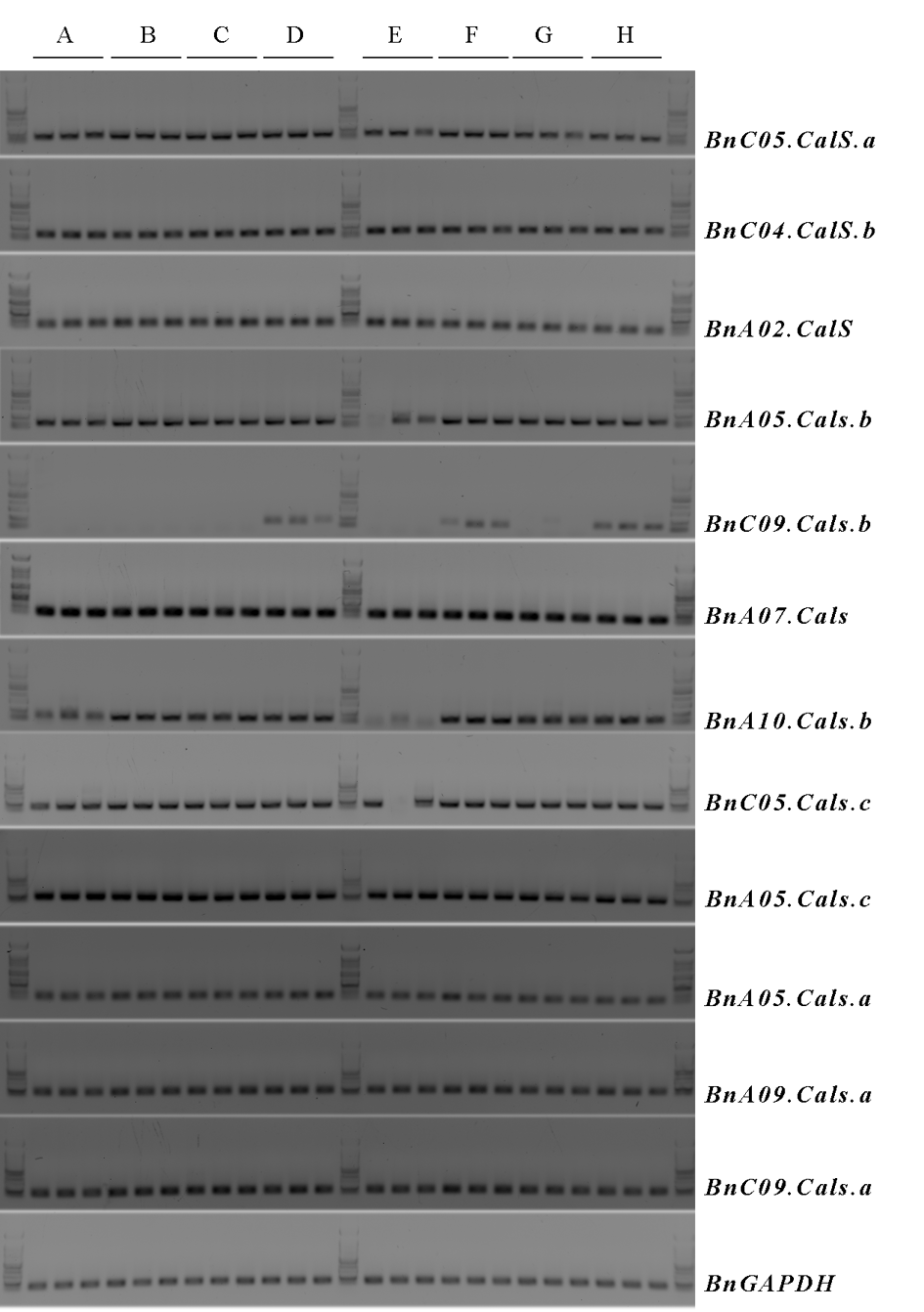

Supplement: Supplementary file 1 [file ijms-19-03769-s001.zip › Figure S4.tif]
